# Investigating the influencing factors of evidence-based healthcare practice adoption

**DOI:** 10.4102/curationis.v47i1.2586

**Published:** 2024-09-12

**Authors:** Lovemore Motsi

**Affiliations:** 1 School of Computing, College of Science, Engineering and Technology, University of South Africa, Pretoria, South Africa

**Keywords:** Perceived ease of use, perceived usefulness, healthcare professionals, relative advantage, government laws and regulations, organisational readiness, evidence-based healthcare practice

## Abstract

**Background:**

In healthcare facilities, evidence-based healthcare practice (EBHP) is becoming more widely acknowledged as a critical element of patient care delivery. An increasingly important component of EBHP is the implementation of electronic health records (EHRs).

**Objectives:**

This study aims to investigate factors that influence EBHP adoption in public healthcare institutions in South Africa.

**Method:**

Four hundred and fifty patients were self-administered to healthcare professionals at an academic public hospital in Gauteng and used in this study. A total of 300 responses were available for use in the final analysis following the data cleaning procedure. Utilising structural equation modelling (SEM), the collected data were analysed.

**Results:**

Perceived ease of use (PEOU) and perceived usefulness (PU) were found to be major variables in the adoption of EBHP along with technological, organisational and environmental factors. The technology context relative advantage (RELA) was shown to have a positive significant influence on the adoption of evidence-based healthcare practice by the PEOU and PU, with the environmental context government laws and regulations (GLRS) and organisational context organisational readiness (ORGR) coming in second and third, respectively.

**Conclusion:**

Perceived ease of use, PU, ORGR, and GLRS are regarded as a vital variables in the implementation of EBHP in South African public hospitals.

**Contribution:**

The study’s conclusions would be helpful to policymakers as they redefine nursing practice. Furthermore, the findings heighten the consciousness of healthcare practitioners regarding the significance of employing evidence-based practice while making decisions.

## Introduction

Hospital technology has changed significantly and globally during the last 20 years (Ben Arfi, Ben Nasr, Khvatova & Ben Zaied 2021; Martínez-Caro et al. 2018). In order to fulfil the needs and expectations of patients, hospitals, in their capacity as healthcare providers, must be able to comprehend, embrace and apply technological developments. Numerous technological advancements aimed at streamlining the healthcare delivery process for customers and healthcare providers have lately been introduced in the health industry. Electronic health records (EHRs) or electronic medical records are among these advancements. According to Alotaibi and Federico (2017), this invention has improved adherence to practice guidelines among healthcare workers and decreased prescription errors and adverse drug responses. Health care information systems, for example, can lower costs associated with providing healthcare services by preventing the ordering of unnecessary tests and procedures by providing patients’ information electronically (Odekunle 2016). Therefore, in the modern world, these advancements have proven essential to raising patient safety and health quality.

Previous studies have examined the factors influencing the adoption of EHRs in healthcare facilities. Using the most recent data to inform healthcare decisions is known as evidence-based practice or EBHP (Lam & Schubert 2019). Anoushiravani and Nunley (2017), suggest that integrating patient records might significantly reduce unnecessary duplication of care and services and have a favourable impact on the nation’s healthcare budget. However, little study has been conducted on the use of EHR as a platform for evidence-based healthcare practices (EBHP). Technology acceptance model (TAM) and technological-organisational-environmental theory (TOE) provide the main theoretical foundation for this paradigm. It is clear that the TAM and TOE models play a vital role in the explanation of technology adoption. Their level of individual explanatory power is constrained, though. The TAM model is sometimes criticised for not incorporating external variables. Other criticisms focus on the model’s triviality, lack of practical value and insufficient ability to explain and predict IT adoption (Gangwar & Raaot 2014). Furthermore, it is estimated that the TAM characteristics account for only 40% of the variation in people’s intentions to use a certain technology (Awa, Ojiabo & Emecheta 2015; Gangwar & Raaot 2014). Technology acceptance model is therefore regarded as a partial model that explains the adoption of IT and other models of technology adoption that are ‘related to human and social change processes, and to the adoption of the innovation model’ should be added to it (Awa et al. 2015).

Now focusing on TOE, it is said that the model is too general and contains vague and confusing constructs; therefore, it should be supplemented with more specific constructs (Awa et al. 2015). It is therefore reasonable to guarantee that the organisational level will be incorporated into the TAM and TOE framework to mitigate their respective limits and bring about a higher explanatory level of understanding IT adoption. Abdekhoda, Dehnad and Zarei (2019) provide evidence in support of this, demonstrating that the integrated TAM–TOE model explained 68% of the variance in EMR uptake. Bryan and Zuva (2021) assert that while TOE incorporates organisational-level technical, environmental and organisational factors, TAM comprises the fundamental external variables that influence an individual’s accepting behaviour. The undefined external variables of the TAM and the unclear major constructs in the TOE framework (Wang, Wang & Yang 2010) were criticised, and the integrated TAM–TOE model was developed to address these issues.

### Conceptual framework

**FIGURE 1:**
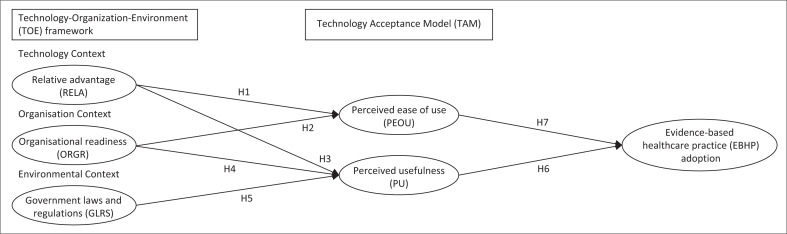
Research model of the study.

The proposed conceptual framework, which uses an integrated TAM–TOE model to identify the determinants influencing the adoption of EBHP, is depicted in Figure 1. To achieve this, the TAM model and TOE framework were both utilised in the model’s development. The three constructs from TOE framework that are most likely to have an impact on the implementation of EHRs, which act as catalysts for the adoption of EBHP, are: (1) technology context (relative advantage [RELA]), (2) organisation context (organisational readiness [ORGR]) and (3) environment context (government laws and regulations [GLRS]). Perceived usefulness (PU) and perceived ease of use (PEOU), two TAM constructs, were used.

#### Technology context

Wang, Gao & Zhou (2022) state that the term ‘technology context refers to the firm’s perceptions of existing and emerging characteristics of the innovative technology’. The technological setting has an impact on the employees’ internal technology expertise. Knowing an organisation’s technology perspective might help determine if it is prepared to accept new technological developments. Three variables of the technology context that the researcher concentrates on are relative benefit, compatibility and complexity. The construct relative advantage was adopted in this study. Relative advantage, according to Pateli, Mylonas and Spyrou (2020), is the degree to which an invention performs better than its alternatives. Furthermore, electronic health record systems facilitate prompt information transfers throughout organisations (Ayaad et al. 2019). All patient and historical data can now be saved on servers because of EHR technologies, which have increased digital information interchange and storage capacities. Relative advantage is important for innovation adoption, according to several studies in the field (Ahmad et al. 2018; AlSharji, Ahmad & Abu Bakar 2018; Effendi, Sugandini & Istanto 2020; Pervin & Sarker 2021). Thus, the following hypothesis is proposed:

**H1:** Relative advantage of electronic health records will influence PEOU of the technology.

**H2:** Relative advantage of electronic health records will influence ease of use of the technology.

#### Organisation context

The organisational context considers the attributes of the company and its assets, such as the degree of staff members’ proficiency with technology, the managerial structure, the quantity of resource slack and the size of the company (Upadhyay & Kumar 2020). One factor make up the organisational context: ORGR. The capacity of the organisation to fully profit from the implementation of electronic health records depends on its willingness to take on all of the risks and problems that come along with the new solutions (Arndt et al. 2017). One of research studies defined ORGR as having the financial and technological means to adopt innovations (Abed 2020). Thus, the following hypothesis is proposed:

**H3:** Organisational readiness of adopting electronic health records will influence PEOU of the technology.

**H4:** Organisational readiness of adopting electronic health records will influence perceived usefulness (PU) of the technology.

#### Environmental context

The competitive pressure, trading partners’ preparedness, sociocultural concerns, government encouragement and technology support infrastructures like access to qualified ICT consultants are all significant aspects of the environmental context, according to Hock-Doepgen et al. (2021). These barriers could affect an organisation’s perception of a certain technology and, in the end, greatly influenced the decision of the organisation to either embrace or reject the technology. Dias (2020) defines a policy as a deliberate plan of action intended to direct choices and achieve wise results. One of the factors influencing the adoption of e-government is the policy issue (Apleni & Smuts 2020). This is because of the fact that electronic health records system implementation and use necessitate a range of laws to control electronic activity. Even though new laws and regulations are seen as innovations, adoption procedures can be interrupted, stopped or need to be redesigned. The suggested study model includes policy and regulations, as they were identified by Haini et al. (2020), Khurshid et al. (2020) and Mustapa et al. (2019) as a key hurdle in prior literature. Thus, the following hypothesis is proposed:

**H5:** Government laws and regulations of adopting electronic health records will influence PU of the technology.

#### Perceived usefulness

Perceived usefulness is the idea that a system can help someone perform better at work in an organisational context (Davis 1989; Zuniarti et al. 2021). When an organisation feels that deploying a particular technology innovation could improve its performance, that organisation is more likely to accept that innovation (Davis 1989). Previous studies have shown that PU has a positive and considerable impact on the rate at which technology is adopted (Fayad & Paper 2015). Most people agree that PU increases the acceptability of technologies (Campbell et al. 2017; Renny, Guritno & Siringoringo 2016). However, PU was not significant in predicting intention to use (Ma & Chan 2014). Hence, according to Campbell et al. (2017), Gunawan and Huarng (2015), and Renny et al. (2016), PU is more important than usability. Previous study has proven the relationship between PU and adoption under different conditions (Erkan & Evans 2016; Tseng & Wang 2016). One advantage of implementing EHR systems has been mentioned: real-time patient monitoring (Ayaad et al. 2020). Thus, the following hypothesis is proposed:

**H6:** Perceived usefulness of electronic health records will influence evidence-based healthcare practice adoption.

#### Perceived ease of use

Perceived ease of use is a person’s estimate of how simple a system will be to use (Eze, Obichukwu & Kesharwani 2021). Perceived ease of use has an impact on PU, according to the TAM model, as users are more likely to judge easy-to-use technology to be advantageous (Chiu & Ku 2015). Electronic health record systems offer the ability to quickly discover and extract patient information, saving healthcare professionals from having to search through files for specific information (Li et al. 2020). Similar to this, a patient’s information is updated instantly and can be instantly shared with other departments or even outside organisations anytime a patient receives treatment, or an allergy is discovered (Li et al. 2020). It appears that every study (Davis 1989; Wahab et al. 2016) agrees that when the PEOU grows, so does technological acceptability. According to Ma and Chan (2014), the only variable now utilised to predict intention to use is PEOU. Previous studies (Fayad & Paper 2015; Almaiah et al. 2021) have shown that technology adoption is significantly and favourably influenced by PEOU. Thus, the following hypothesis is proposed:

**H7:** Perceived ease of use of electronic health records will influence evidence-based healthcare practice adoption.

## Research methods and design

### Data collection

Convenience sampling was used in this investigation. According to Gaille (2020), the convenience sampling technique is the process of gathering data from people in the population who are easily accessible to do so. The researcher considers variables including accessibility, closeness, desire to engage in the study and ability to offer necessary information for the research study to establish the ‘convenience’ of the sample. Healthcare professionals at an academic public hospital in Gauteng province provided information via a self-administered questionnaire. Medical healthcare professionals such as medical doctors, nurses, pharmacists, radiographers and the like are typically not considered medical professionals, also referred to as medical healthcare professionals. According to Montero-Marin et al. (2014), these professionals include doctors, nurses, veterinarians, medical assistants, dentists, midwives, physiotherapists, optometrists, dermatologists, cardiologists, practitioners and therapists among many more.

Three hundred and fifty of the 450 received questionnaires, which the researcher administered were used in this study. There was insufficient information for 150 of them to be considered appropriate for data analysis. The data cleaning process yielded a total of 300 responses were used in the final analysis. Prior to distributing the questionnaire to every respondent, a pilot study was conducted with 40 medical professionals who were specifically chosen from a District Hospital in Tshwane, Gauteng province. The questionnaire was adjusted based on the feedback and recommendations from the pilot study before the main study began. The study was a cross-sectional design and implemented between 12 June 2021 and 30 August 2021.

### Demographic information of the sample

A total of 211 (70.4%) participants were females and 89 (29.6%) were males. Out of all the participants, 102 (34.0%) were aged between 31 years and 40 years, and 26 (8.6%) were younger than 25 years. Of the participants, 98 (32.6%) were aged between 21 years and 50 years, 57 (19.0%) were aged between 41 years and 50 years and 17 (5.6%) were aged above 50 years. Nurses accounted for 243 (81.0%) of the total, while medical practitioners made for 16 (5.3%). Of those with experience, 147 (49.0%) had 6 years to 10 years’ worth, 68 (27.6%) had 2 years to 5 years’ worth, 76 (25.3%) had 10 or more years’ worth and 19 (0.6%) had less than a year’s worth of experience. Table 1 presents the demographic data for the sample values.

**TABLE 1:** The demographic information of the sample.

Demographics	Item	Frequency (*n*)	%
Gender	Male	89	29.6
	Female	211	70.4
Age (years)	Less than 25	26	8.6
	25–30	98	32.6
	31–40	102	34.0
	41–50	57	19.0
	More than 50	17	5.6
Occupation	Medical doctor	16	5.3
	Pharmacist	12	4.0
	Radiology	10	3.3
	Physiotherapist	9	3.0
	Nurse	243	81.0
	Dentist	10	2.6
Work experience (years)	Less than 1	19	0.6
	2–5	68	27.6
	6–10	147	49.0
	More than 10	76	25.3

### Data analysis and measurement

An appropriate instrument development is necessary to ascertain the true correlations between constructs. On the one hand, interrater dependability of instrument results must be obtained, and on the other, internal consistency of measuring instruments must be guaranteed. An integrated TAM and TOE framework is used in this study to examine the variables that affect to investigate what influences South African public hospitals’ adoption of evidence-based healthcare practices. The data were initially examined in Statistical Package for Social Sciences (SPSS) version 25 using multicollinearity analysis and descriptive statistics. Then, structural equation modelling, or SEM, was used to evaluate the measurement items and structural models. Six modified indicators from sources (Fortes & Rita 2016) were used to quantify mistreatment using the trust variable in three dimensions. The scales and items for TAM and TOE were chosen based on prior research and literature.

In information systems (IS) and information technology (IT) adoption research, most of the constructs included in this study have measuring items that have been suggested and/or verified. However, some modifications were required to guarantee that these items were appropriate for the study’s context. Perceived usefulness and PEOU have five and six constructs’ items, respectively, and were adopted from Kwak, Seo and Ahn (2022) and Davis (1989). The dependent variable containing five construct items, evidence-based healthcare practice (EBHP), was borrowed from Motsi (2023). In addition, three constructs with six constructs each were adopted from Boonsiritomachai (2014); Tornatzky and Fleischer (1990); Yeh, Lee and Pai (2014): the environment context (GLRS), the organisation context (ORGR) and the technology context (relative advantage). In order to measure the items, a 5-point Likert scale on an interval range from ‘strongly disagree’ to ‘strongly agree’ was developed specifically for this study. The questionnaire was validated by six senior academicians who were experts on the subject matter.

### The goodness of fitness

For testing each model, commonly used fit indices were employed (Kenny 2020). Among them are popular indices like the Tucker-Lewis index (TLI), normed fit index (NFI), incremental fit index (IFI), adjusted goodness of fit index (AGFI) and goodness of fit index (GFI). Using AMOS (analysis of moment structures), confirmatory factor analysis (CFA) is carried out to verify and validate the measurement that was employed. ‘AMOS is an IBM SPSS Statistics module intended for path analysis, confirmatory factor analysis (CFA), and structural equation modelling (SEM) analysis of covariance structure models’ (Sarstedt, Ringle & Hair 2021). Table 2 presents the goodness of fit (GOF) values.

**TABLE 2:** Goodness of fit.

Measures of GOF	Threshold	Estimate	Measurement results
CMIN/*df*	Between 1 and 3	1.927	Good Fit
*df*	> 0.90	0.902	Acceptable
RMSEA	< 0.06	0.047	Good Fit
SRMR	< 0.08	0.056	Good Fit
PClose	> 0.05	0.905	Good Fit

CMIN, Chi-square minimum; *df*, degrees of freedom; GOF, goodness of fit; RMSEA, root mean square error of approximation; SRMR, Standardised root mean squared; PClose, test of close fit for the model in SEM; SEM, structural equation modelling.

### Measurement model

The AMOS SEM output was used following the goodness of fitness test, and the results are consistent with Ferdinand (2018). If the construct responsibleness worth (CR) of a mensuration model is less than 0, then the composite responsibleness of the model is assumed to be sensible responsibleness to live every hidden variable. In addition, the average variance extracted (AVE) has a value greater than 0 if it is 7 or more than 5, which is acceptable. Between 0.5 and 0.6 in the exploration responsibleness analysis will be accepted, and at the importance level (*p*) 0.05 (5%), a unidirectional check is assigned in SEM AMOS with a confidence level of 95%.

The commonly used vital worth (CR) > 1.96 indicates that the acceptance in normalcy is rejected. Table 3 presents the construct reliability and variance extracted values.

**TABLE 3:** Construct reliability and variance extracted.

Constructs	Item code	Factor loadings	Error	CR	AVE
Relative advantage	-	-	-	0.84	0.52
	RELA1	0.80	0.60	-	-
	RELA2	0.74	0.62	-	-
	RELA3	0.72	0.61	-	-
	RELA4	0.78	0.57	-	-
	RELA5	0.82	0.59	-	-
	RELA6	0.80	0.50		
Organisational readiness	-	-	-	0.82	0.50
	ORGR1	0.81	0.51	-	-
	ORGR2	0.78	0.53	-	-
	ORGR3	0.80	0.52	-	-
	ORGR4	0.81	0.64	-	-
	ORGR5	0.75	0.67	-	-
	ORGR6	0.70	0.52		
Government laws and regulations	-	-	-	0.86	0.54
	GLRS1	0.81	0.65	-	-
	GLRS2	0.78	0.62	-	-
	GLRS3	0.74	0.57	-	-
	GLRS4	0.82	0.61	-	-
	GLRS5	0.80	0.62	-	-
	GLRS6	0.78	0.68		
Perceived ease of use	-	-	-	0.84	0.62
	PEOU1	0.74	0.61	-	-
	PEOU2	0.78	0.60	-	-
	PEOU3	0.80	0.53	-	-
	PEOU4	0.73	0.62	-	-
	PEOU5	0.78	0.65	-	-
	PEOU6	0.79	0.67		
Perceived usefulness	PU1	0.81	0.51	0.82	0.50
	PU2	0.78	0.53	-	-
	PU3	0.80	0.52	-	-
	PU4	0.81	0.64	-	-
	PU5	0.75	0.52	-	-
Evidence-based healthcare practice adoption	-	-	-	0.84	0.62
	EBHPA1	0.74	0.61	-	-
	EBHPA2	0.78	0.60	-	-
	EBHPA3	0.80	0.53	-	-
	EBHPA4	0.79	0.63	-	-
	EBHPA5	0.73	0.65		

AVE, average variance extracted; CR, construct responsibleness; EBHPA, evidence-based healthcare practice adoption; GLRS, government laws and regulations; ORGR, organisational readiness; PEU, perceived ease of use; PU, perceived usefulness; RELA, relative advantage.

The findings highlighted in Table 3 demonstrate the validity and reliability of the 26 indicators, which are supported by quantity (CR) of at least 0.7 and the average variance extracted (AVE) greater than 0.05. As a result, it is common knowledge that every indicator used in this research is legitimate and trustworthy, and it can be used for analysis. The testing results for the many hypotheses that were postulated in Figure 1 and then investigated in this study are summarised in Table 4.

**TABLE 4:** Summary of testing hypotheses.

H	Path	β	T-statistics	*p*	Result
H1	RELA→PEU	0.117	1.974	0.049	Supported
H2	RELA→PU	0.198	2.003	0.046	Supported
H3	ORGR→PEU	0.232	3.557	0.000	Supported
H4	ORGR→PU	0.188	2.116	0.035	Supported
H5	GLRS→PU	0.191	2.595	0.010	Supported
H6	PU→EBHPA	−0.102	1.983	0.048	Supported
H7	PEU→EBHPA	0.496	8.808	0.000	Supported

EBHPA, evidence-based healthcare practice adoption; EHR, electronic health record; GLRS, government laws and regulations; H, hypothesis; ORGR, organisational readiness; PEU, perceived ease of use; PU, perceived usefulness; RELA, relative advantage.

### Ethical considerations

Ethical clearance to conduct this study was obtained from the UNISA-CAES Health Research Ethics Committee (reference number 2019/CAES/075). The Deputy Director of Clinical Services, an academic public hospital in Gauteng province, gave their approval for this study to be conducted. Study’s objectives and procedures were explained to the participants, and participation was completely voluntary. The submission of a completed questionnaire served as proof of consent. All replies were kept anonymous; no names or other personally identifiable information was required.

## Results and discussion

Using an integrated TAM-TOE model, the purpose of this descriptive study was to examine the variables that affect the adoption of evidence-based healthcare practices. The findings showed that healthcare professionals who took part in this study have a positive perception of both TAM constructs – the practicality and usability of EHRs. Another significant problem that health care organisations face, according to the literature, is preventative care (Ayaad et al. 2020). More specifically, it is expected to greatly enhance patient health outcomes by expanding preventative care. Perceived usefulness and ease of use (PEU) were significantly positively impacted by relative advantage (RELA→PEU: β = 0.117, *p* < 0.05; RELA→PU: β = 0.198, *p* < 0.05). The results of this study support Davis’s (1989) assertion that user desire to adopt and use new technology is significantly influenced by how easy and useful the technology is to use. According to a study on health professionals’ acceptance of health information technology, EHR adoption was significantly influenced by its simplicity of use (Abd-alrazaq et al. 2019). Perceived ease of use was a major factor in the adoption of EHR in other studies (Hossain et al. 2019; Kavandi et al. 2020).

The results showed that, in terms of the government rules and regulations, it influences both the adoption and the use of electronic health records. Perceived usefulness and ease of use are both significantly impacted (GLRS→PU: β = 0.191, *p* < 0.05). This outcome is in line with earlier research (De Pietro 2018; Wani & Maholtra 2018), which showed that to comply with established industry norms and laws, healthcare organisations have to change their record-keeping and sharing procedures as well as their healthcare practices. The results show that EHR systems, which adhere to industry norms and regulations, have come to play a major role in health care organisations. Kamatula (2018) discovered that the effective execution of e-government and e-records management requires the establishment of simplified strategies, laws, rules and regulations. This means that having written regulations to direct actions is crucial for efficient e-records management and the ultimate deployment of evidence-based healthcare practice. According to ORGR→PEU: β = 0.232, *p* < 0.05; ORGR→PU: β = 0.188, *p* < 0.05, PU and ease of use are positively correlated with ORGR. The findings of this study are in line with most of the earlier research (Vakola 2014), which noted that while conducting a change effort, there is an increasing interest in developing employee preparedness for change. These results corroborated the work of other researchers who have studied change and its causes; yet the majority of these studies concentrate on readiness for change from an organisational or management standpoint (Weiner 2020; Ahmad et al. 2021).

Adoption of evidence-based healthcare practice is influenced by PU and perceived ease of use (PEU→EBHPA: β = 0.496, *p* < 0.05; PU→EBHPA: β = –0.102, *p* < 0.05). This outcome is in line with other research by (Ayaad et al. 2020), which found that EHR systems raise patient safety and improve the quality of patient care particularly through increases in operational efficiency and decreases in mistakes. Moreover, EHR frequently encourages improved institutions and interorganisational collaboration. Electronic health record systems encourage more information exchange and interorganisational coordination.

### Recommendations

This study has certain limitations that offer the potential for more research in the future. Firstly, this study employed a convenience sample of physicians and other healthcare providers who worked in a single public hospital. The results from this one hospital cannot be extrapolated to all public hospitals in other provinces of South Africa because there are multiple public hospitals in the Gauteng province. Secondly, to ensure more robust results, the model should be evaluated on a greater number of samples, even though the study’s sample can be deemed statistically appropriate. Subsequent research endeavours ought to examine the diverse array of elements that impact EBHPD, including but not limited to trust, barriers, time efficiency and stakeholder considerations. This may result in the development of new dimensions or construct by which healthcare providers in public hospitals can assess the effectiveness of EBHPD. Future models could incorporate the use of qualitative research methodologies to investigate different types of variables in order to improve the understanding of the factors influencing EBHPD. Thirdly, in order to meaningfully direct resources towards shaping the contextual elements that have a significant impact on implementation outcomes, it is necessary to have a deeper understanding of the interactions between leadership and other critical aspects linked to implementation success.

## Conclusion

Findings of this study show that the use of EHRs, which serve as accelerators for the adoption of EBHPD, is discussed in this integrative study. From TOE, one variable represents the technological context (RELA), one variable represents the environment context (GLRS), and one variable represents the organisation context (ORGR). Two TAM constructs, PU and PEOU, have been identified as key determinants influencing evidence-based healthcare practices (EBHPD). Implementation efforts are probably supported by making use of these characteristics.

Organisational contextual features interact, impact, modify, promote or hinder the EBHPD and its implementation efforts. This is supported by the finding that these features synergistically influenced implementation efforts, indicating that context is more than just a physical setting or backdrop for implementation. The research contributes to theory as well as practice. According to the findings, the measuring scales employed to assess the EBHPD, and its antecedents are accurate and dependable. A suggested model has been verified, confirming the relationship between GLRS, PU, PEOU, ORGR and RELA. Study results on the association between adoption and EHRs align with the findings of (Ayaad et al. 2020), which indicate that EHRs have a significant impact on EBHPD.
